# Survival trends of patients with oral and oropharyngeal cancer treated at a cancer center in São Paulo, Brazil

**DOI:** 10.6061/clinics/2020/e1507

**Published:** 2020-04-06

**Authors:** Luiz Paulo Kowalski, Max Moura de Oliveira, Rossana Veronica Mendoza Lopez, Diego Rodrigues Mendonça e Silva, Mauro Kazuo Ikeda, Maria Paula Curado

**Affiliations:** I Departamento de Cirurgia de Cabeca e Pescoco e Otorrinolaringologia, A.C. Camargo Cancer Center, Sao Paulo, SP, BR; II Grupo de Epidemiologia e Estatistica em Cancer, Centro Internacional de Pesquisa, A.C. Camargo Cancer Center, Sao Paulo, SP, BR; III Centro de Investigacao Translacional em Oncologia, Institute of Cancer do Estado de Sao Paulo, Sao Paulo, SP, BR

**Keywords:** Survival Analysis, Prognosis, Oral Cavity, Oropharynx, Cancer

## Abstract

**OBJECTIVE:**

We aimed to estimate the overall survival (OS) and conditional survival (CS) in patients diagnosed with oral and oropharyngeal squamous cell carcinoma (SCC) and to determine their survival trends.

**METHODS:**

The study included all consecutive patients treated at the A.C. Camargo Cancer Center for oral or oropharyngeal SCC between 2001 and 2012. Data were obtained from the Hospital Cancer Registry. OS and CS were analyzed using the Kaplan-Meier method to evaluate the probability of survival with Cox predictor models.

**RESULTS:**

Data of 505 oral and 380 oropharyngeal SCC patients obtained in 2001–2006 and 2007–2012 were analyzed. Most of the oral SCC (59%) and oropharyngeal SCC (90%) patients had stages III–IV SCC. The 5-year OS for patients with oral SCC was 51.7%, with no significant difference between the first and second periods. The CS rates in 2007–2012 were 65% after the first year and 86% up to the fifth year. For oropharyngeal SCC, the 5-year OS rate was 45.0% in the first period. The survival rate increased to 49.1% from 2007 to 2012, with a reduction in the risk of death (HR=0.69;0.52–09.2). The CS estimates from 2007 to 2012 were 59% after the first year and 75% up to the fifth year.

**CONCLUSION:**

Survival across the two time periods remained stable for oral SCC but showed a significant increase for oropharyngeal SCC, possibly because of improvements in the patients' response to radiotherapy, such as intensity-modulated radiation therapy, and the use of more accurate diagnostic imaging approaches.

## INTRODUCTION

In 2018, 354,864 new cases of lip and oral cancer (OC) and 92,877 new cases of oropharyngeal cancer were reported worldwide. The number of deaths from lip cancer and OC was 51,005, whereas that from oropharyngeal cancer was 51,005 1. In 2018, 19,898 new cases of lip cancer and OC were reported in Latin America and Caribbean countries 1, whereas 14,700 new cases were reported in Brazil 2. A total of 9,180 individuals from Latin America and Caribbean countries and 4,629 from Brazil developed oropharyngeal cancer 1. Therefore, the number of individuals diagnosed with oral and oropharyngeal squamous cell carcinoma (SCC) in Brazil is almost half the number of those diagnosed with oral and oropharyngeal SCC in Latin America and Caribbean countries.

Cancers in the oral cavity and oropharynx are classified as head and neck cancers, and 90% of them are SCC 3,4. Tobacco and alcohol use are the main risk factors for OC. For oropharyngeal cancer, human papilloma virus (HPV) infection has also been considered a risk factor and prognostic biomarker 5-7.

The survival rates for head and neck cancer vary depending on the site involved 7. Previous European and American population-based studies reported that the 5-year relative survival of German people with OC diagnosed in 2002–2006 was 54.6% 8, whereas that of Danish people with head and neck cancer was 62.4% (2010–2014) 9. For American individuals with cancer in the oral cavity, oropharynx, and subsites (base of tongue, hard palate, tonsil, etc.), SEER reported a 5-year relative survival of 62.9% 4.

In the United States (US), the 5-year relative survival of oropharyngeal cancer patients increased from 33.3% in 1992–1996 to 42.2% in 2002–2006 4.

On the basis of the hospital data, there were differences in overall survival (OS) rates between public and private institutions. In Argentina, the survival estimate for OC was 39% in public hospitals 10. In Chile, the survival estimate for OC was 46% in private hospitals 11. In Brazil, Santa Catarina reported a 5-year OS for OC and pharyngeal cancer of 33.3% 12. In Maceio, northeast of Brazil, the 5-year OS for oropharyngeal cancer was 27.8% 12,13. In Santa Maria, south of Brazil, the 5-year OS for OC and oropharyngeal cancer was 42%; however, patients with oropharyngeal cancer had the worst survival rates 14.

Retrospective studies have shown survival differences according to tumor site and subsite (base of tongue, hard palate, and tonsil) for oropharyngeal cancer and sex and age for OC 15.

Conditional survival (CS) provides an estimate of the chances of surviving in the second to the fifth year after diagnosis, after surviving for at least a year; it is a dynamic survival indicator wherein the prognosis is based on the number of years survived after treatment 16,17. Thus, we evaluated the survival of patients with OC and oropharyngeal cancer by assessing their OS and CS rates in order to provide prognostic information. CS analysis provide prognostic information for cancer survivors; it was performed to estimate the probability of survival after 1 to 5 years 17. In the US, a marked improvement in the CS of oropharyngeal cancer patients (26.6%–60.0%) was observed during the period 1997–1998 18.

The present study aimed to estimate the OS and CS in patients diagnosed with oral and oropharyngeal SCC at the A.C. Camargo Cancer Center (ACCCC) in Sao Paulo, Brazil, to determine the progress in patients' survival. The study was conducted in H&N patients treated at a specialized treatment center between 2001 and 2012.

## METHODS

Data of patients diagnosed with oral SCC (C02, C05 except C051-C052, and C06) and oropharyngeal SCC (C01, C051-C052, C09, and C10) according ICD-O3 between 2001 and 2012 were extracted from the Hospital Cancer Registry of ACCCC 19; the morphological codes (8050/3, 8070/3, 8074/3, 8076/3, and 8083/3) 19 for oral and oropharyngeal SCC were registered at the ACCCC with follow-up until December 31, 2017. The Ethics Committee approved the study (n° 2462/17 on Dec 5, 2017). The patients were stratified by age group (≤50 years, 51–60 years, and >60 years); sex (male and female), diagnostic period (2001–2006 and 2007–2012), clinical stage (I–II and III–IV) 20,21 and treatment procedure (surgery alone, surgery with chemoradiation, and other/palliative care). The absolute and relative frequencies were described.

OS was calculated from the date of diagnosis to the date of death (of any cause) or the date on which the latest information was available. Survival curves were calculated using the Kaplan-Meier method; the probabilities for survival at 1 year, 3 years, and 5 years were described using independent variables. The differences in survival curves were compared using a log-rank test. The hazard ratio (HR) as its corresponding 95% confidence interval (CI) were determined using a Cox regression model.

We calculated the CS, which is the dynamic probability of the patient surviving *t* years, given that a patient has already survived for *s* years after diagnosis 22. Thus, we were able to estimate the patient's chance of surviving an additional *t* years after surviving for *s* years (with the total being *t+s* years). CS was calculated on the basis of survival probability data accumulated using the Kaplan-Meier method according to the following formula:



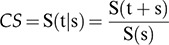



CS rates were plotted for 1–5 years after diagnosis. The level of significance was 5% for all hypotheses. All analyses were performed using SPSS v.25 for Windows.

## RESULTS

Between 2001 and 2012, 885 patients with oral and oropharyngeal SCC were treated at ACCCC. Of them, 505 had OC, whereas 380 had oropharyngeal cancer. In relation to age distribution, 50% of patients with OC were aged >60 years, whereas 42% of those with oropharyngeal cancer were aged. With regard to sex, OC (2:1) and oropharyngeal cancer (6:1) were more common in men. The majority of patients had advanced-stage (stage III–IV) cancer (OC: 59% (290/505), oropharyngeal cancer: 90% (339/380); Table 1). The proportions of patients with missing 5-year clinical follow-up data were 6.4% (OC) and 3.9% (oropharyngeal cancer).

### Oral SCC

There was an improvement in the treatment (combining surgery and chemoradiotherapy) response of stage III–IV patients, which increased from 48.7% in 2001–2006 to 63.2% in 2007–2012 (*p*<0.006) (Table 2).

**Figure 1:**
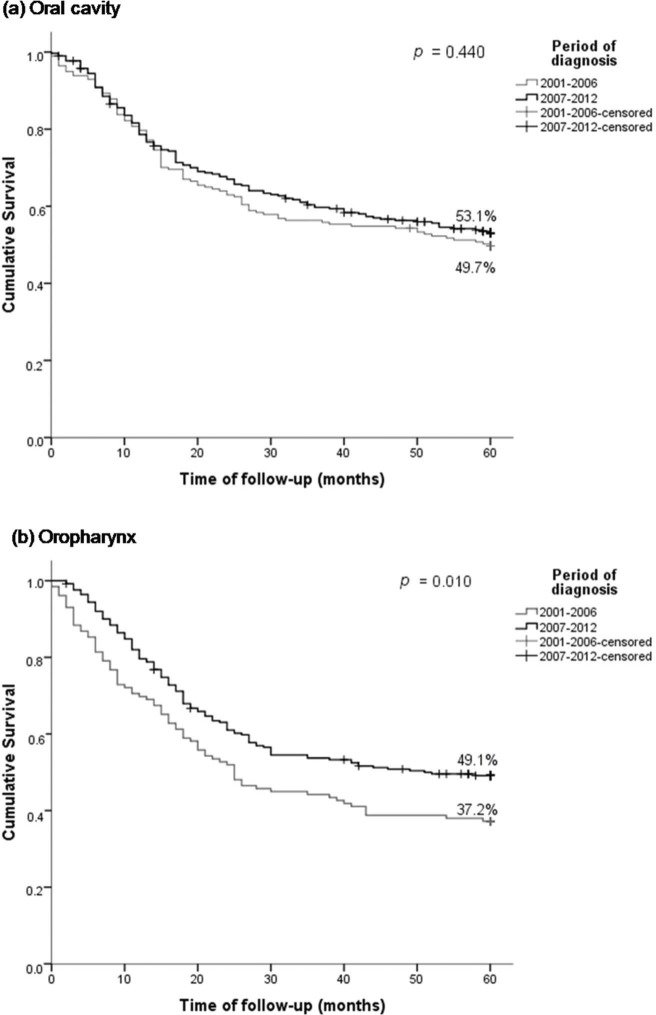
Overall survival of patients with oral and oropharyngeal squamous cell carcinoma treated at the A. C. Camargo Cancer Center, Hospital Cancer Registry, 2001–2012, by period of diagnosis.

The OS rates for OC were 79.0%, 58.4%, and 51.7% in the first, third, and fifth years after diagnosis, respectively. The OS for oral SCC in 2001–2006 was 49.7% and that in 2007–2012 was 53.1% (*p*=0.440) (Figure 1a). The 5-year OS was higher in patients aged ≤50 years (61.8%) and in those with stage I–II cancer (74.0%) (Table 3). The risk of death was 1.42 times higher in patients with oral SCC aged >60 years and 3.58 higher in those with clinical stage III–IV SCC (Table 4).

**Figure 2:**
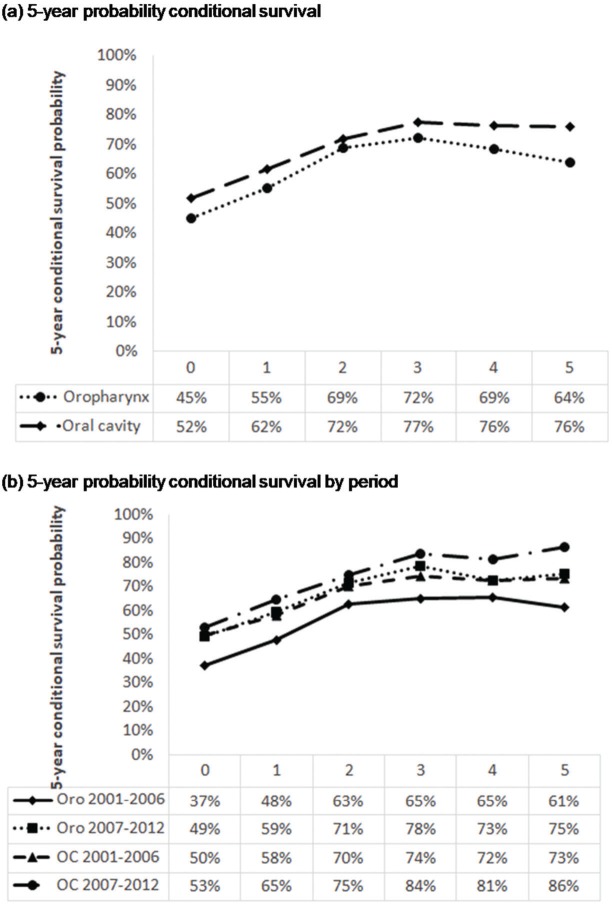
Five-year probability conditional survival of patients with oral and oropharyngeal squamous cell carcinoma treated at the A. C. Camargo Cancer Center, Hospital Cancer Registry 2001–2012, according period of diagnosis. Oro, oropharynx; OC, oral cavity.

CS of patients with oral SCC increased from 65% after the first year to 86% in the fifth year after diagnosis, demonstrating an improvement in survival in the most recent period (Figure 2b). With the passage of time, the chances of survival increase. However, the risk of death from oral SCC remained unchanged in the two study periods.

### Oropharyngeal SCC

Approximately 75.7% of patients with oropharyngeal SCC were treated with chemoradiation, with differences noted between the first and second period (*p*<0.001). Of the patients with stage III–IV cancer treated between 2001 and 2006, 31% received chemoradiation; between 2007 and 2012, 71.7% of the patients received chemoradiation (Table 2). OS probabilities for oropharyngeal SCC were 76.3% and 45.0% in the first and fifth years after diagnosis, respectively. The 5-year OS was higher in patients diagnosed between 2007 and 2012 (49.1%; *p*=0.010) (Figure 1b), those aged ≤50 years (58.1%; *p*=0.007), and those with stage I–II cancer (75.2%; *p*<0.001) (Table 3). The HR in the adjusted analysis and risk of death were higher in patients aged >60 years (HR 1.88) and those with stage III–IV disease (HR: 3.23) (Table 4).

The 5-year CS after having survived for 1 year was 48% for those diagnosed between 2001 and 2006 and 59% for those diagnosed between 2007 and 2012 (Figure 2b). Therefore, the survival rate of oropharyngeal SCC increased in the more recent period.

## DISCUSSION

The 5-year OS rates were 52% for oral SCC and 45% for oropharyngeal cancer in the period 2001–2012. However, the 5-year survival of patients with oral SCC remained unchanged (49.7%–53.1%), whereas that of patients with oropharyngeal SCC increased from 37.2% to 49.1%. By contrast, Ridder et al. reported a 5-year survival rate of 60% 15, whereas Du et al. 23 reported 5-year survival rates of 69% and 56% for OC and oropharyngeal cancer, respectively.

The majority of patients with oral SCC in our study were aged 60 years, whereas those with oropharyngeal SCC tended to be younger, which is similar to the findings in the literature 24. A previous study conducted in ACCCC identified that comorbidities are prognostic factors for older adults with head and neck cancer 25. A review by VanderWale et al. 26 described that the treatment toxicity can be quite morbid for older adults as they are often excluded from clinical trials that provide definitions on the standards of care.

In both OC and oropharyngeal cancer, we observed an increase in mortality risk for patients aged >60 years and those with stage III/IV SCC, which is similar to the findings reported by Ridder et al. 15. Although a greater mortality risk was reported in men 15,21,23,24, this study did not find a survival difference between sexes.

The 5-year OS for stage I–II patients with OC and oropharyngeal cancer was around 74%, although the proportion of patients with early-stage (I–II) OC was 41% and that of patients with early-stage (I–II) oropharyngeal cancer was 9.8%. Zanoni et al. 27 reported an OS rate of 64.4% (1985–2015) for OC patients admitted at the Memorial Sloan Kettering Cancer Center, a rate higher than that observed in this study; however, 58% of the patients treated had stage I–II SCC, whereas 39% had stage III–IV SCC. In 11 hospitals in Europe, 45% of the OC patients had stage III–IV SCC 15. Du et al. 23 observed that 38% of OC patients had stage III–IV SCC, whereas 59% had stage III–IV SCC. Amit et al. 28, in a multicentric international study conducted in seven international institutions including ACCCC in two decades (1990 to 2011), reported an improvement (from 59% to 70%) in the survival of patients with OC in the most recent period.

The multicenter CHANCE study (USA) reported a 54% 5-year OS for OC 21, which is similar to our data. In Portugal, Monteiro et al. 29 observed that 71% of their patients with OC were diagnosed at stage III–IV, with a survival rate of 41%, without significant improvements over time (38% for 2000–2004 and 42% for 2005–2009). In a university hospital in Santa Maria, Brazil, the OS was 42% in 2004–2014 14. Thus, the OS rate for patients with OC has been around 55% at most treatment centers.

Survival for oropharyngeal cancer improved significantly between 2001 and 2012 (from 37.2% to 49.1%), although around 90% of the treated patients had stage III–IV cancers. The presence of HPV in the oropharynx and its role as a prognostic factor affected the therapeutic planning in these cases and had an influence on the improvement in survival 5. Brazilian studies showed that the majority of patients diagnosed with oropharyngeal cancer are not HPV-positive (i.e., low prevalence, 3%–9%) 30,31.

A review on the treatment of older patients with head and neck cancer indicated that oropharyngeal cancer patients aged 65 years and older have survival outcomes similar to those of younger patients with head and neck cancer; however, they may experience worse toxicity 26. Sandulache et al. 32 reported a 40% 5-year OS for oropharyngeal cancer and indicated that 30% of the patients were unable to complete the treatment.

For locally advanced oropharyngeal cancer, chemotherapy combined with radiation treatment has been reported to significantly improve OS 33,34. Du et al. 23 observed that 84% of oropharyngeal cancer patients had stage III–IV cancers, which is the same as the finding of this study (90%). Monteiro et al. 29 reported a 27% 5-year OS rate for oropharyngeal cancer, with a significant reduction in mortality from 2000–2004 (28%) to 2005–2009 (24%) in Northern Portugal. Patients with advanced stages have worst survival 35. Although 90% of the patients with oropharyngeal cancer treated at the ACCCC had clinical stage III–IV cancers, the survival among these patients increased from the previous period to the more current period, likely because of the changes in therapeutic planning, such as the use of IMRT and new target therapies 36.

For oropharyngeal cancer, there was a difference in terms of the period of diagnosis, with a lower risk of death observed in patients treated in the more recent period (2007–2012), similar to the findings of Monteiro et al. 29.

With regard to the 5-year CS of OC patients, CS increased to 17% in patients who survived 1 year and to 13% in those who survived 5 years after the diagnosis (2007–2012 *versus* 2001–2006); similar results were observed in a single institution in the same period 37.

For oropharyngeal cancer, a similar increase in CS was observed in 2007–2012 and 2001-2006; the probability of CS at the first year was 11%, and it increased to 14% after 5 years. The CS of oral cancer patients 5 years after diagnosis had increased, whereas that of patients with oropharyngeal cancer had decreased. The decrease in oropharyngeal cancer survival after 5 years remains unclear.

The lack of progress in CS among older adults with oral and oropharyngeal SCC could be related to the presence of comorbidities and other competitive causes of death. In this article, because of the number of patients, we were not able to stratify patients by age, stage, and period to estimate the CS as Yang et al. 17 described.

The OS of patients with OC and oropharyngeal cancer increased from 2007 to 2012; however, the majority of patients who underwent treatment had advanced-stage cancer. The OS of patients with stage I and II oral SCC was 74%, whereas that of oropharyngeal SCC patients was 75.2%, which is two times higher than the rates observed among those with stage III and IV cancers with these topographies.

Hospital Cancer Registries are database that were created under the law 38 in Brazil, but the use of these registries has limitations: they cannot provide comprehensive clinical information and data on patients' outcomes. At ACCCC, the Hospital Cancer Registry is in collaboration with Fundação Oncocentro de São Paulo; from 2000 to 2012, the registry had 36,343 registered cancer cases 39.

The limitation of this study is the lack of information on the p16 status for oropharyngeal SCC because this biomarker had not yet been routinely adopted during the study period and the cause of death was not described in the Hospital Cancer Registry.

Therefore, early diagnosis of OC and oropharyngeal cancer is likely to increase the chances of cure and survival among these patients.

## AUTHOR CONTRIBUTIONS

Kowalski LP and Curado MP contributed to the conception and design. Oliveira MM and Lopez RVM contributed to the analysis and interpretation of data, and manuscript drafting. Silva DRM extracted the data and drafted the manuscript. Kowalski LP and Ikeda MK critically reviewed the manuscript. Curado MP discussed and critically reviewed the manuscript and supervised the research. All authors approved the final version of the manuscript to be published.

## Figures and Tables

**Table 1:** Characteristics of patients with oral and oropharyngeal SCC treated at the A.C. Camargo Cancer Center during the diagnostic period, HCR, 2001–2012.

	Oral cavity	Oropharynx	Total
	N=505	N=380	N=885
Characteristics	n (%)	n (%)	n (%)
Age group (yrs)			
≤50	118 (23.4)	82 (21.6)	200 (22.6)
51-60	139 (27.5)	138 (36.3)	277 (31.3)
>60	248 (49.1)	160 (42.1)	408 (46.1)
Gender			
Male	335 (66.3)	324 (85.3)	659 (74.5)
Female	170 (33.7)	56 (14.7)	226 (25.5)
Period of diagnosis			
2001-2006	197 (39.0)	129 (33.9)	326 (36.8)
2007-2012	308 (61.0)	251 (66.1)	559 (63.2)
Clinical stage			
I-II	203 (41.2)	37 (9.8)	240 (27.6)
III-IV	290 (58.8)	339 (90.2)	629 (72.4)

SCC, squamous cell carcinoma; HCR, Hospital Cancer Registry.

**Table 2:** Treatment adopted for patients with oral and oropharyngeal squamous cell carcinoma at the A.C. Camargo Cancer Center, according to period of diagnosis and clinical stage, HCR, 2001–2012.

		Period of diagnosis			
Clinical stage	Treatment	2001-2006	2007-2012	Total	*p*
**Oral cavity**					
I-II	Surgery only	60 (80)	100 (78.1)	160 (78.8)	0.494
	Surgery with (C)RT	13 (17.3)	24 (18.8)	37 (18.2)	
	(C)RT	1 (1.3)	4 (3.1)	5 (2.5)	
	Palliative	1 (1.3)	0 (0)	1 (0.5)	
	Subtotal	75 (100)	128 (100)	203 (100)	
III-IV	Surgery only	45 (37.8)	34 (19.9)	79 (27.2)	0.006
	Surgery with (C)RT	58 (48.7)	108 (63.2)	166 (57.2)	
	(C)RT	11 (9.2)	24 (14)	35 (12.1)	
	Palliative	5 (4.2)	5 (2.9)	10 (3.4)	
	Subtotal	119 (100)	171 (100)	290 (100)	
Total	Surgery only	107 (54.3)	139 (45.1)	246 (48.7)	0.087
	Surgery with (C)RT	71 (36)	134 (43.5)	205 (40.6)	
	(C)RT	12 (6.1)	29 (9.4)	41 (8.1)	
	Palliative	7 (3.6)	6 (1.9)	13 (2.6)	
	Total	197 (100)	308 (100)	505 (100)	
**Oropharynx**					
I-II	Surgery with or without (C)RT	10 (83.3)	18 (72.0)	28 (75.7)	0.452
	(C)RT	2 (16.7)	7 (28.0)	9 (24.3)	
	Subtotal	12 (100)	25 (100.0)	37 (100.0)	
III-IV	Surgery with or without (C)RT	71 (61.2)	57 (25.6)	128 (37.8)	<0.001
	(C)RT	36 (31.0)	160 (71.7)	196 (57.8)	
	Palliative	9 (7.8)	6 (2.7)	15 (4.4)	
	Subtotal	116 (100.0)	223 (100.0)	339 (100.0)	
Total	Surgery with or without (C)RT	81 (62.8)	77 (30.7)	158 (41.6)	<0.001
	(C)RT	39 (30.2)	167 (66.5)	206 (54.2)	
	Palliative	9 (7.0)	7 (2.8)	16 (4.2)	
	Total	129 (100.0)	251 (100.0)	380 (100.0)	

(C)RT, (chemo)radiotherapy; HCR, Hospital Cancer Registry.

**Table 3:** Survival probabilities at 1, 3, and 5 years in patients with oral and oropharyngeal squamous cell carcinoma treated at the A.C. Camargo Cancer Center, HCR, 2001–2012.

		Oral cavity					Oropharynx				
			Probability of survival					Probability of survival			
Variable	Group	Deaths/total	1 year	3 years	5 years	*p*	Deaths/total	1 years	3 years	5 years	*p*
Overall		240/505	79.0%	58.4%	51.7%		207/380	76.3%	50.4%	45.0%	
Period	2001-2006	99/197	79.7%	56.3%	49.7%	0.440	81/129	69.8%	44.2%	37.2%	0.010
	2007-2012	141/308	78.6%	59.7%	53.1%		126/251	79.6%	53.7%	49.1%	
Gender	Male	166/335	79.5%	55.9%	49.6%	0.185	178/324	76.5%	50.4%	44.7%	0.894
	Female	74/170	78.1%	63.3%	56.0%		29/56	75.0%	51.1%	47.2%	
Age group (yrs*)*	≤50	45/118	85.6%	65.3%	61.8%	0.017	34/82	82.9%	61.9%	58.1%	0.007
	51-60	64/139	84.1%	60.1%	53.2%		72/138	72.5%	52.9%	47.7%	
	>60	131/248	73.1%	54.1%	46.0%		101/160	76.1%	42.4%	35.9%	
Clinical stage	I-II	51/203	93.5%	83.9%	74.0%	<0.001	9/37	94.6%	78.0%	75.2%	<0.001
	III-IV	184/290	69.6%	40.8%	36.2%		195/339	74.6%	47.8%	42.0%	

HCR, Hospital Cancer Registry.

**Table 4:** Hazard ratio associated with 5-year survival in patients with oral and oropharyngeal squamous cell carcinomas treated at the A.C. Camargo Cancer Center, Hospital Cancer Registry, 2001–2012.

		Oral cavity				Oropharynx			
Variable	Group	HR_unadjusted_ (95%CI)	*p* value	HR_adjusted_ (95%CI)	*p* value	HR_unadjusted_ (95%CI)	*p* value	HR_adjusted_ (95%CI)	*p* value
Period	2001-2006	1.00		1.00		1.00		1.00	
	2007-2012	0.90 (0.70-1.17)	0.443	0.91 (0.70-1.18)	0.487	0.70 (0.53-0.92)	0.012	0.69 (0.52-0.92)	0.012
Gender	Female	1.00		1.00		1.00		1.00	
	Male	1.20 (0.91-1.58)	0.189	1.14 (0.86-1.51)	0.360	1.03 (0.69-1.52)	0.885	0.94 (0.63-1.39)	0.747
Age group (yrs)	≤50	1.00		1.00		1.00		1.00	
	51-60	1.27 (0.87-1.87)	0.213	1.05 (0.71-1.55)	0.807	1.36 (0.91-2.05)	0.136	1.46 (0.97-2.20)	0.073
	>60	1.60 (1.14-2.24)	0.007	1.42 (1.01-2.01)	0.044	1.80 (1.22-2.65)	0.003	1.88 (1.27-2.78)	0.002
Clinical stage	I-II	1.00		1.00		1.00		1.00	
	III-IV	3.66 (2.68-5.00)	<0.001	3.58 (2.61-4.89)	<0.001	3.10 (1.59-6.04)	0.001	3.23 (1.65-6.30)	0.001

*HR, hazard ratio.
